# Minimally Invasive Derotational Osteotomy of Long Bones: Smartphone Application Used to Improve the Accuracy of Correction

**DOI:** 10.3390/jcm12041335

**Published:** 2023-02-07

**Authors:** Chang-Wug Oh, Kyeong-Hyeon Park, Joon-Woo Kim, Dong-Hyun Kim, Il Seo, Jin-Han Lee, Ji-Wan Kim, Sung-Hyuk Yoon

**Affiliations:** 1 Department of Orthopedic Surgery, School of Medicine, Kyungpook National University, Kyungpook National University Hospital, Daegu 41944, Republic of Korea; 2 Department of Orthopedic Surgery, Severance Children’s Hospital, Yonsei University College of Medicine, Seoul 03722, Republic of Korea; 3 Department of Orthopedic Surgery, Asan Medical Center, University of Ulsan College of Medicine, Seoul 05505, Republic of Korea

**Keywords:** derotational osteotomy, malrotation, rotational malalignment, smartphone application

## Abstract

Correction of rotational malalignments caused by fractures is essential as it may cause pain and gait disturbances. This study evaluated the intraoperative use of a smartphone application (SP app) to measure the extent of corrective rotation in patients treated using minimally invasive derotational osteotomy. Intraoperatively, two parallel 5 mm Schanz pins were placed above and below the fractured/injured site, and derotation was performed manually after percutaneous osteotomy. A protractor SP app was used intraoperatively to measure the angle between the two Schanz pins (angle-SP). Intramedullary nailing or minimally invasive plate osteosynthesis was performed after derotation, and computerized tomography (CT) scans were used to assess the angle of correction postoperatively (angle-CT). The accuracy of rotational correction was assessed by comparing angle-SP and angle-CT. The mean preoperative rotational difference observed was 22.1°, while the mean angle-SP and angle-CT were 21.6° and 21.3°, respectively. A significant positive correlation between angle-SP and angle-CT was observed, and 18 out of 19 patients exhibited complete healing within 17.7 weeks (1 patient exhibited nonunion). These findings suggest that using an SP app during minimally invasive derotational osteotomy can result in accurate correction of malrotation of long bones in a reproducible manner. Therefore, SP technology with integrated gyroscope function represents a suitable alternative for determination of the magnitude of rotational correction when performing corrective osteotomy.

## 1. Introduction

Intramedullary (IM) nailing and minimally invasive plate osteosynthesis (MIPO) are the most commonly used treatment measures for diaphyseal and metaphyseal fractures of long bones, respectively [1,2]. These closed-reduction surgical techniques provide better treatment outcomes (e.g., excellent fracture healing and rapid patient recovery) compared to open reduction with internal fixation, as they require smaller incisions and minimal soft tissue dissection. Malrotation, a common complication of IM nailing or MIPO, is often overlooked in comparison to angular deformities of the coronal and sagittal planes. Postoperative malrotations exceeding 10° have been observed in 50% of patients with femoral and tibial fractures [3,4,5,6], and restoration of the alignment is essential as abnormal loading may lead to pain, instability, and early degeneration [7].

Although challenging, a variety of osteotomy procedures have been used previously for the correction of rotational malalignment. During open osteotomy, the surgeon marks reference lines over the bone and/or uses angle templates to visually determine the desired correction intraoperatively [8]. This traditional technique can disturb bone healing and increase blood loss through extensile exposure, and this risk can be decreased by using minimally invasive corrective osteotomy techniques with fluoroscopic guidance [9,10]. Although intraoperative C-arm images have been used for objective measurement of the lesser trochanter profile, cortical step sign, and diameter difference sign [11,12], visual fluoroscopic estimation may prove to be imprecise when determining the angle of rotation [13].

Although the gyroscopic function of smartphone (SP) technology has previously been used to measure angles in bone models simulating rotational deformities [14,15], there is limited evidence of its efficacy in measuring the rotational angle during surgical corrective osteotomy. Therefore, in the current study, a consecutive series of derotational osteotomies were performed using an SP application (SP app) intraoperatively, and a comparison of the desired angle, measured using the SP app, and the corrected angle, measured using a postoperative CT scan, was carried out. The hypothesis being tested was that the proposed osteotomy technique using an SP app would achieve accurate correction of rotational deformity in a reproducible manner.

## 2. Materials and Methods

This study was approved by the ethics committee of the institute, and informed consent was collected from all participants prior to commencement of this study and after provision of relevant verbal and written information. Patients diagnosed with malunion/nonunion and rotational malalignment secondary to surgical intervention for femoral or tibial fractures were selected to undergo the proposed procedure. A thorough evaluation of the diagnosis was carried out to identify any additional requirements during the surgical procedure.

The indication is based on symptoms and clinical and radiological evaluation. No clear indications for surgical correction are reported in the literature. Patients were included if they met any of the following criteria:

1. Malrotation of more than 15 degrees after fracture surgery;
2. Malrotation greater than 10 degrees in nonunion patients;
3. Among patients with malrotation of more than 15 degrees and symptomatic patellofemoral malalignment interfering with daily life.

Patients were excluded if the patient refused additional surgery or had asymptomatic malrotation.

**Figure 1:**
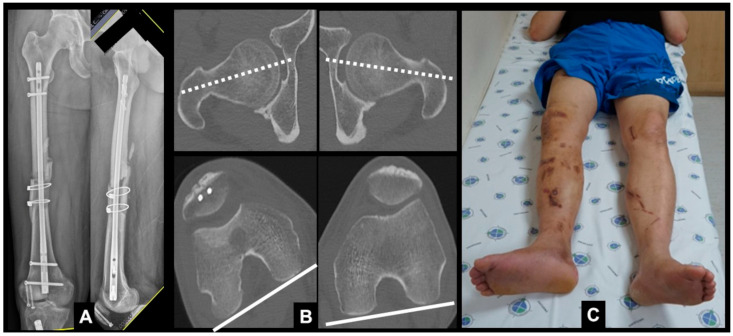
(**A**) Preoperative anteroposterior and lateral radiographs showing nonunion after retrograde nailing; (**B**) CT scan showing an evident difference in the femoral torsional angle between the affected and unaffected sides; (**C**) externally rotated foot on the right side indicating retroversion of the femur.

In addition to standard radiographic evaluation, all patients underwent preoperative computerized tomography (CT) scans to allow accurate identification of anatomical deformities. Femoral torsion was determined by measuring the angle between the long axis of the femoral neck and a line drawn parallel to the dorsal aspect of the femoral condyles on an axial CT image [16] (Figure 1). Tibial torsion was defined as the angle between the posterior tibial axis of the proximal tibia and the bimalleolar axis of the distal tibia on an axial CT image [17]. The difference in the angle of rotational alignment of the affected and contralateral unaffected limbs was calculated.

### 2.1. Surgical Technique

Preoperative evaluation included calculation of the required angulation of rotational correction and identification of any additional deformities. The surgical plan included derotational osteotomy at the previously fractured area and use of either an IM nail or plate for fixation, selected based on the anatomical location of the pre-existing implant.

**Figure 2:**
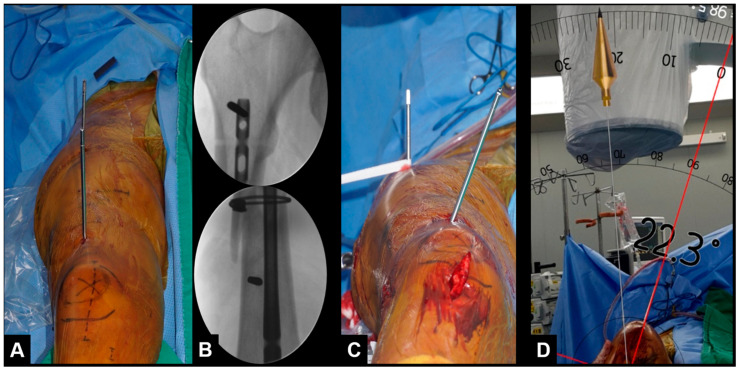
(**A**,**B**) Two parallel Schanz pins placed above and below the nonunion without interfering with the nail; (**C**) internal rotation of the distal segment after removal of the previous nail; (**D**) the smartphone application measured a 22° correction.

During surgery, the patient was placed in a supine or lateral position on a radiolucent table, and their whole lower extremity was draped. Prior to commencement of the osteotomy, two parallel 5 mm Schanz pins were carefully placed above and below the previous fracture such that they did not interfere with the pre-existing or new implants (nail or plate) to allow accurate measurement of the correction (Figure 2).

**Figure 3:**
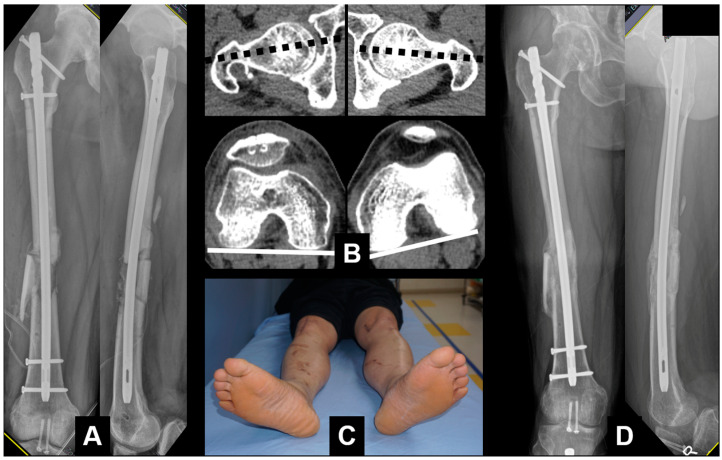
(**A**) Postoperative radiographs showing antegrade nailing with bone graft at the nonunion site; (**B**) CT scan showing similar angles of anteversion; (**C**) similar rotation in foot position; (**D**) complete healing observed 1 year postoperatively.

**Figure 4:**
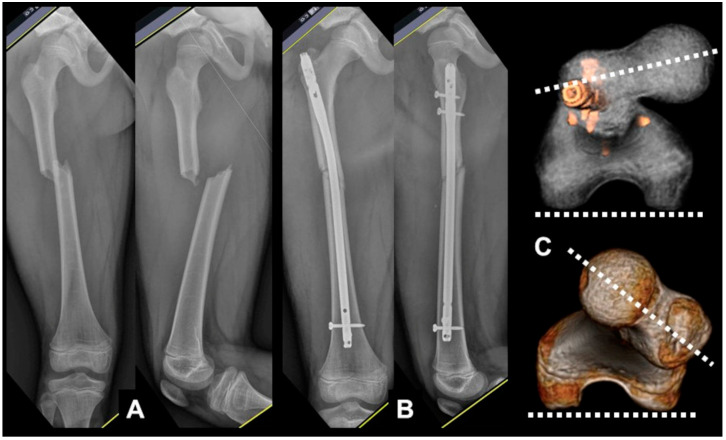
(**A**) An 11-year-old male patient diagnosed with a femoral-shaft fracture; (**B**) IM nailing was carried out; (**C**) postoperative CT scan showing the decreased angle of anteversion compared with the noninjured side.

**Figure 5:**
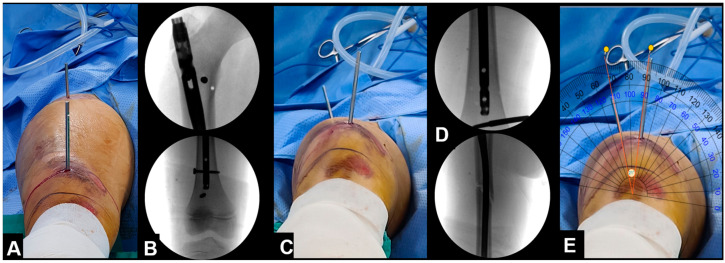
(**A**,**B**) Parallel Schanz pins placed in the proximal and distal femur; (**C**,**D**) internal rotation of the distal segment after removal of the distal interlocking screws; (**E**) the smartphone application measured a 20° correction.

**Figure 6:**
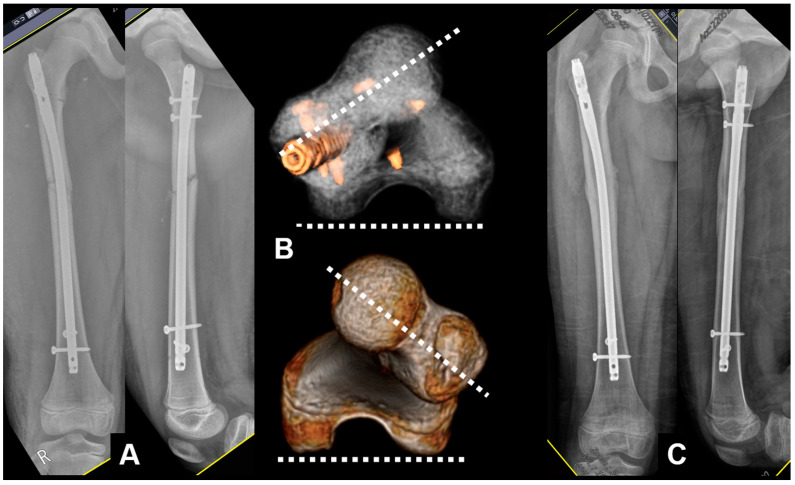
(**A**) Postoperative radiographs showing revised fixation of distal interlocking; (**B**) CT scan showing similar angles of anteversion; (**C**) complete healing observed 6 months postoperatively.

**Figure 7:**
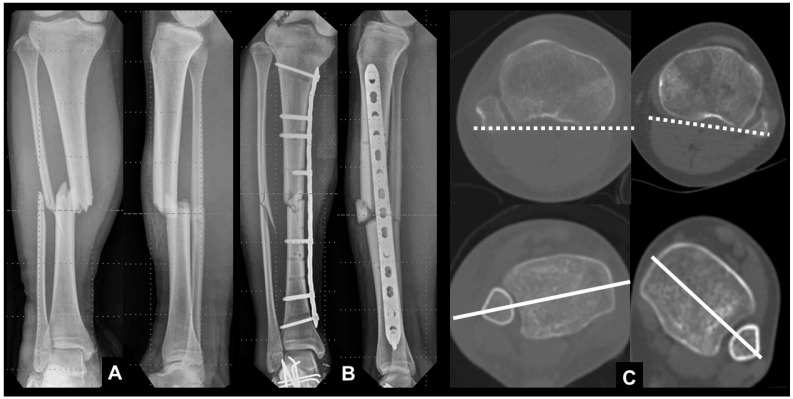
(**A**) A 15-year-old girl with a tibia shaft fracture; (**B**) minimally invasive plate osteosynthesis was carried out; (**C**) postoperative CT scan showing a moderate difference (over 16 degrees) in rotation compared with the noninjured side.

**Figure 8:**
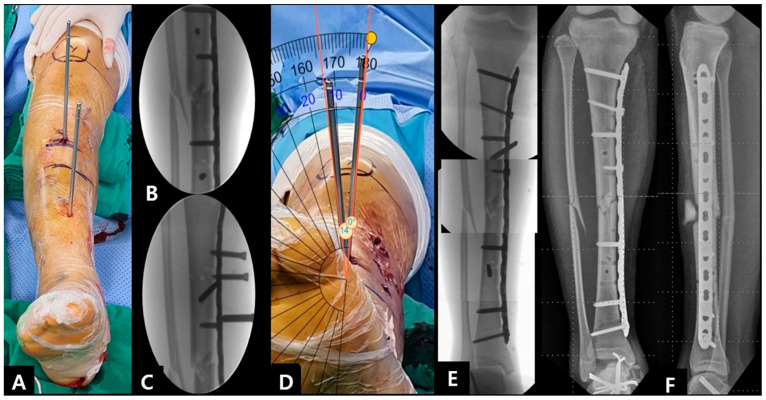
(**A**,**B**) Parallel Schanz pins placed in the proximal and distal tibia; (**C**) screws were removed at the proximal segment; (**D**) external rotation of the distal segment and the smartphone application measured about 14° correction; (**E**) screws were fixed at the proximal segment; (**F**) postoperative radiographs showing the improved alignment after revision procedure.

**Figure 9:**
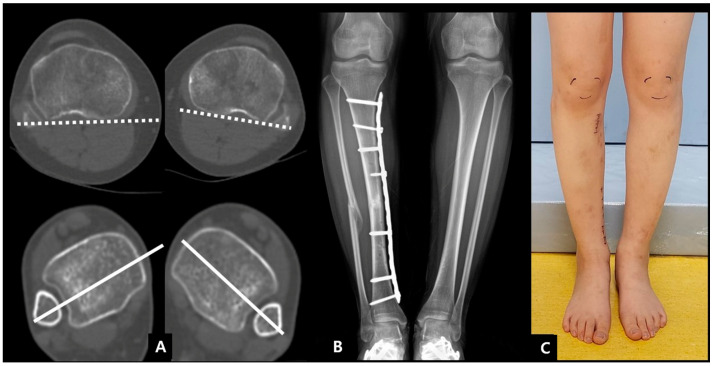
(**A**) Postoperative CT scan showing similar angles of rotation; (**B**,**C**) complete healing with the similar alignment of lower leg was observed 1 year postoperatively.

Thereafter, the pre-existing implant placed during the earlier osteosynthesis was removed, and in patients with nonunion or malunion, percutaneous osteotomy was performed at the planned site. Minimally invasive osteotomy was performed using a 1–2 cm incision and multiaxial drilling with C-arm control, and the final procedure was completed by connecting the multiple drill holes using a half-inch osteotome (Figure 3). In patients with postoperative malalignment, the proximal or distal fixation was disassembled without removing the full implant (Figure 4, Figure 5, Figure 6, Figure 7, Figure 8 and Figure 9).

After reaming the medullary canal, a new IM nail was inserted, and derotation was carried out by manually rotating the distal part of the limb. The extent of rotation was estimated by measuring the angle between the two Schanz pins. The intraoperative angle of correction was measured by an assistant standing at the end of the table using a free protractor SP app (angle-SP). The corrective osteotomy aimed to achieve an angle of correction equivalent to the rotational alignment of the contralateral side, determined using a preoperative CT scan. A maximum difference of 5° between the measured and target values was considered acceptable. Distal fixation was then performed while maintaining the rotational correction, and the Schanz pins were removed. Clinical examination was performed after removal of the drapes to confirm rotational correction, and the patient was then sent to the recovery room.

### 2.2. Smart Phone Application

The SP application (SP app) used in this study was the Smart Protractor application (Smart Tools Co., Dae-gu, South Korea). Measurement with the SP app is obtained by positioning a virtual protractor, visible on the SP screen, on photography obtained using the SP camera. The assistant takes a photo of the Schanz pins, saves it, measures the angles, and observes the value. The picture should be taken with the camera positioned along the imaginary line between two pins. After the photo has been taken and saved, two red lines appear on the screen. The lines can be dragged across the screen to place the virtual goniometer on the axis of the Schanz pins, finely adjusting them until the goniometer is positioned correctly. Pictures judged subjectively wrong by the surgeon because of a perspective error can be deleted. The angle-SP measurement was repeated three times, and the average value was determined.

### 2.3. Postoperative Care and Evaluation

Postsurgical clinical evaluation of rotational correction involved comparison of foot rotation and internal and external rotation of the hip joint between the affected and unaffected limbs while the patient was still in the supine position on the table. Based on the patient’s level of tolerance, range of motion was resumed slowly, and all patients were allowed use of partial weight-bearing crutches.

Postoperative CT scans were used to compare the angles (angle-CT), and a maximum difference of 5° was considered acceptable. The correlation between angle-SP (measured intraoperatively with the SP app) and angle-CT (measured postoperatively using a CT scan) was assessed.

Clinical follow-up was carried out 1, 2, and 3 months postoperatively, and every 3 months thereafter until bone union was achieved. Radiographic evaluation was carried out at each visit, and bone union was defined as the presence of an appropriate bridging callus and resolution of persistent fracture lines in at least 3 out of 4 radiographic views. All patient radiographs were assessed by two independent board-certified orthopedic surgeons.

### 2.4. Statistical Analysis

Paired *t*-test and Pearson’s correlation coefficient tests were used to compare the angle-SP and angle-CT, and all statistical analyses were performed using the IBM SPSS software, version 19.0 (IBM Co., Armonk, NY, USA). A *p*-value < 0.05 was considered statistically significant.

## 3. Results

Between March 2013 and February 2021, of the 31 identified patients who underwent derotational osteotomy in the femur or tibia, 19 patients treated with minimally invasive osteotomy with the SP app were included. Among 19 cases (17 cases of the femur, 2 cases of the tibia), 17 cases were treated using IM nailing, and 2 cases using MIPO. All patients had a minimum follow-up of 1 year. The mean age of the patients at the time of surgery was 37.9 years (range: 11–77 years).

Four patients were diagnosed with acute postoperative malalignment, 15 patients with late manifestation (nonunion *n* = 12; malunion, *n* = 3), 11 patients with externally rotated deformities, and 8 patients with internally rotated deformities. Preoperative CT scans showed a mean difference of 22.3° (range: 11.2°–38.3°) in rotational angle. Among those exhibiting nonunion, four patients presented with additional angular malalignments that were simultaneously corrected. Fixation was carried out using IM and MIPO in 17 and 2 patients, respectively, and additional bone grafting was carried out in 8 patients. Sixteen patients were operated on in the supine position, while three were in the lateral position.

The mean intraoperative angle-SP was 21.6° (range: 10.2°–36.1°), while the postoperative angle-CT was 21.3° (range: 13.9°–39.2°). The mean difference in angle of rotation was 2.3°, and the variations in value were within the accepted range of 5° (range: −4.2° to +4.8°). A statistically significant positive correlation between angle-SP and angle-CT was observed (Pearson’s correlation coefficient r = 0.972; *p*-value = <0.001).

The mean duration of healing was 17.7 weeks (range: 12–24 weeks) in 18 out of 19 patients, and all of them exhibited acceptable improvement in gait with symmetric angles of foot progression. One patient exhibited nonunion requiring a secondary bone graft, while three patients presented with minor complications related to the Schanz pins (bent pin *n* = 2; broken pin, *n* = 1), which likely occurred during manual derotational correction. None of the patients developed any infections (Table 1).

## 4. Discussion

Rotational osteotomy is a commonly used surgical treatment measure for the correction of malalignment caused by congenital, developmental, or posttraumatic factors. Techniques involving intraoperative fluoroscopic imaging have been used to ensure accurate correction, particularly along the coronal and sagittal planes. However, no reliable methods of assessing the intraoperative rotational alignment of the lower limbs have been reported to date. SP technology has been used previously in various orthopedic surgeries [18,19], with experimental studies examining its accuracy in the measurement of rotational deformities [14,15]. The findings were largely similar to those obtained using CT scanning with markers placed over artificial bones without any soft tissue coverage. However, angle measurement may be simpler in saw bone models as the markers are clearly visible, and this is in contrast to actual bony tissues in the extremities, which are typically enveloped by muscle and soft tissues that can interfere with visualization of the markers and prevent accurate measurement of angles. The findings of this study showed a strong correlation between angle-SP and postoperative angle-CT and, to the best of our knowledge, this is the first clinical series to examine derotational osteotomy and report accurate intraoperative measurement of correction using an SP app with an integrated gyroscope. Therefore, SP-assisted derotational osteotomy may be considered a suitable alternative for traditional osteotomies using open measuring.

Several fixation techniques for acute fractures also use fluoroscopic imaging to accurately measure rotation intraoperatively. They typically use anatomical landmarks (such as the lesser trochanter profile, patellar and fibular position, and native femoral torsion of the hip compared to the posterior femoral condylar plane) on the contralateral unaffected extremity as a template [20,21,22]. While these methods are also applicable in corrective osteotomy, variations in local anatomy may raise uncertainty regarding the accuracy of techniques based on visual estimation [23]. High rates of malrotation have been reported in patients with highly comminuted fractures, pre-existing anatomical deformities, or bilateral injuries [3,5]. Another limitation of techniques using fluoroscopy is the additional operative time required. Intraoperative use of mobile CT scanning [24,25] or computer navigation [26] is considered an ideal method for assessment of rotation, although their use is limited because of high associated costs, increased radiation exposure, and logistical issues in the operating room. In comparison, the proposed technique using an SP app measures the angle of rotation intuitively in real-time, is more economically viable, minimizes radiation exposure, and is also logistically convenient.

A recently introduced innovative surgical technique that corrects femoral malrotation using 3D printing technology [27] has the advantage of using customized cutting guides that allow accurate estimation of the angle of correction of malrotation. Additionally, it also requires shorter operating time and less radiation exposure. However, this technique requires a considerably invasive surgical approach as the 3D printed guides must be fixed proximal and distal to the osteotomy site, thus increasing the risk of blood loss, infection, and disturbed bone healing. Therefore, this technique may be unsuitable for the correction of postsurgical malalignment where the exposure of the previous fracture is unnecessary. In comparison, the proposed technique using SP app-assisted measurement requires minimal surgical exposure for percutaneous osteotomy and can even be carried out without opening the fracture site. Therefore, it allows for a less invasive approach, consequently reducing surgical morbidity and risk of infection. Additionally, it can also promote bone healing, as it allows preservation of the periosteal blood supply and the surrounding soft tissue. In the current study, 18 out of 19 patients exhibited primary bone healing without any infectious complications. Therefore, we believe that the proposed technique for derotational osteotomy sufficiently meets the requirement of minimal invasiveness.

Precise intraoperative measurement of the angle of derotation is important as it contributes to the functional outcomes after corrective osteotomy. Care should be taken to place markers during the procedure in order to allow accurate measurement of this parameter. After osteotomy, intraoperative measurement of the angle between the two markers is essential. A previous study examining version abnormalities of the femur used flat triangular osteotomy templates to estimate the angle between the markers visually [9]. However, its measurement may be rough and inaccurate, since the angles of triangles cannot match the different amount of the rotational malalignment. Although a sterilized goniometer may also be considered a suitable alternative, direct measurement may not be easy based on the distance between the markers. In comparison, the SP app can act as a customized alternative of the virtual goniometer that can determine the magnitude of rotational deformity precisely. This clinical trial showed a maximum difference of <5° between the affected and unaffected sides, suggesting that the use of a contemporary SP app is likely to achieve accuracy of measurement during derotational osteotomy.

Various operative techniques for correcting rotational deformities have been described in the literature. The conventional method is open exposure at the deformity level [28,29]. The correction angle is planned on preoperative CT scans and intraoperatively marked on the bone with Kirschner wires or Schanz screws. Subsequently, a transverse osteotomy and derotation are performed [28]. In our study, minimally invasive derotational osteotomy was carried out. This technique allows closed osteotomy without stripping the surrounding soft tissues, reducing surgical morbidity and risk of infection. The periosteum is left intact, which improves callus formation and bone healing. However, it is difficult to perform accurate angle correction, and there are disadvantages in that radiation exposure and intraoperative time increase. The proposed SP app technique can intuitively measure the rotation angle in real time and precisely determine the rotational deformity’s magnitude. So, it can minimize radiation exposure and also decrease intraoperative time. However, the minimally invasive technique may be technically challenging surgery and requires a steep learning curve.

This study had several limitations, including a small sample size and no comparator groups. Future studies with a larger number of patients and appropriate comparison groups treated using other methods are necessary to confirm the efficacy of using the SP app for this purpose. This is the first study to carry out a large series of derotational osteotomies for the treatment of posttraumatic malalignments only. However, there are several factors that may affect the accuracy of measurement when using this technique. Firstly, errors may occur if the height and direction of the SP are not parallel to the two markers, emphasizing the need to ensure that the camera is placed on an imaginary line that is parallel to the markers when measuring. Secondly, errors may also occur if the markers are bent, with three patients in the current study exhibiting Schanz pins that were bent or broken during manual derotation. In such cases, reinsertion of the markers is essential if the fault is detected prior to measurement, and rotation of the distal limb should be carried out without holding the markers. Thirdly, loss of derotation during distal fixation of the implant can lead to over or undercorrection. An innovative technique using electromagnetic tracking (EMT) to monitor the angle of derotation continuously during surgery has been proposed previously [30]. However, this method is technically difficult and requires sterilization of specific parts (sensors or pointers) prior to use in the surgical setting. In comparison, the SP app does not require any complex equipment, and also offers minimal discrepancies between intraoperative derotation and postoperative results.

## 5. Conclusions

In conclusion, the SP app allows precise assessment of intraoperative angles during derotation osteotomy. SP app may help the newly introduced minimally invasive derotational osteotomy. It is a reliable and reproducible procedure to predict accurate angle measurement and produce excellent bone healing and function.

## Figures and Tables

**Table 1:** Patient demographics and outcomes.

No.	Sex	Age (Year)	Location	Cause	Rotational Deformity	Associated Deformity	Implant	Bone Graft	Pre- Operative Difference	Angle Measured Using SP App	Post- Operative Difference	Gained Angle	Time to Union (Weeks)
1	M	15	Femur	Malalignment	Internal		Nail	none	16.1	15.8	2.3	13.8	12
2	M	37	Femur	Nonunion	External		Nail	YES	38.3	36.1	3.2	35.1	16
3	M	22	Femur	Malalignment	External		Nail	none	22.3	20.9	−1.5	23.8	16
4	M	49	Femur	Malunion	External	Varus	Nail	YES	15.6	14.1	1.4	14.2	nonunion
5	F	32	Femur	Nonunion	Internal		Nail	YES	27.8	29.5	4.3	23.5	18
6	M	50	Femur	Malunion	Internal	Varus	Plate	none	20.2	20.3	−2.1	22.3	16
7	M	57	Femur	Nonunion	External		Nail	none	20	19.2	−4.2	24.2	18
8	M	70	Femur	Nonunion	External		Nail	YES	35.9	35.3	−3.3	39.2	20
9	M	57	Femur	Nonunion	Internal		Nail	YES	23.5	22.2	2.5	21	24
10	M	58	Femur	Nonunion	External		Nail	YES	11.2	10.4	−2.7	13.9	20
11	M	46	Femur	Nonunion	External		Nail	YES	23.9	23.4	4.8	19.1	18
12	M	11	Femur	Malalignment	External		Nail	none	20.6	20.1	−1.6	22.2	14
13	M	27	Femur	Nonunion	Internal		Nail	none	21.8	20.5	4.2	17.6	18
14	M	24	Femur	Malunion	Internal	Varus	Nail	none	17.8	19.6	3.0	14.8	16
15	F	15	Tibia	Malalignment	Internal	Procurvatum	Plate	none	16.3	14.7	1.3	15	14
16	M	77	Femur	Nonunion	External		Nail	none	23.1	20.2	4.6	18.5	20
17	M	17	Femur	Nonunion	External		Nail	YES	17.2	15.9	2.5	14.7	20
18	M	20	Tibia	Malunion	External		Nail	none	30.3	32.5	1.5	28.8	20
19	M	36	Femur	Nonunion	Internal		Nail	none	22.1	19.3	−1.8	23.9	18
		37.9							22.3	21.6	2.3	21.3	17.7

## Data Availability

Source data may be shared by the corresponding author upon reasonable request.
